# Supplementary material for: High-throughput ultrastructure screening using electron microscopy and fluorescent barcoding
Source: J Cell Biol. 2019 Jul 9;218(8):2797–811. doi: 10.1083/jcb.201812081 (PMC6683748; doi:10.1083/jcb.201812081)
Supplement: Supplemental Materials (PDF) [file JCB_201812081_sm.pdf]

Supplemental material

Bykov et al., https://doi.org/10.1083/jcb.201812081

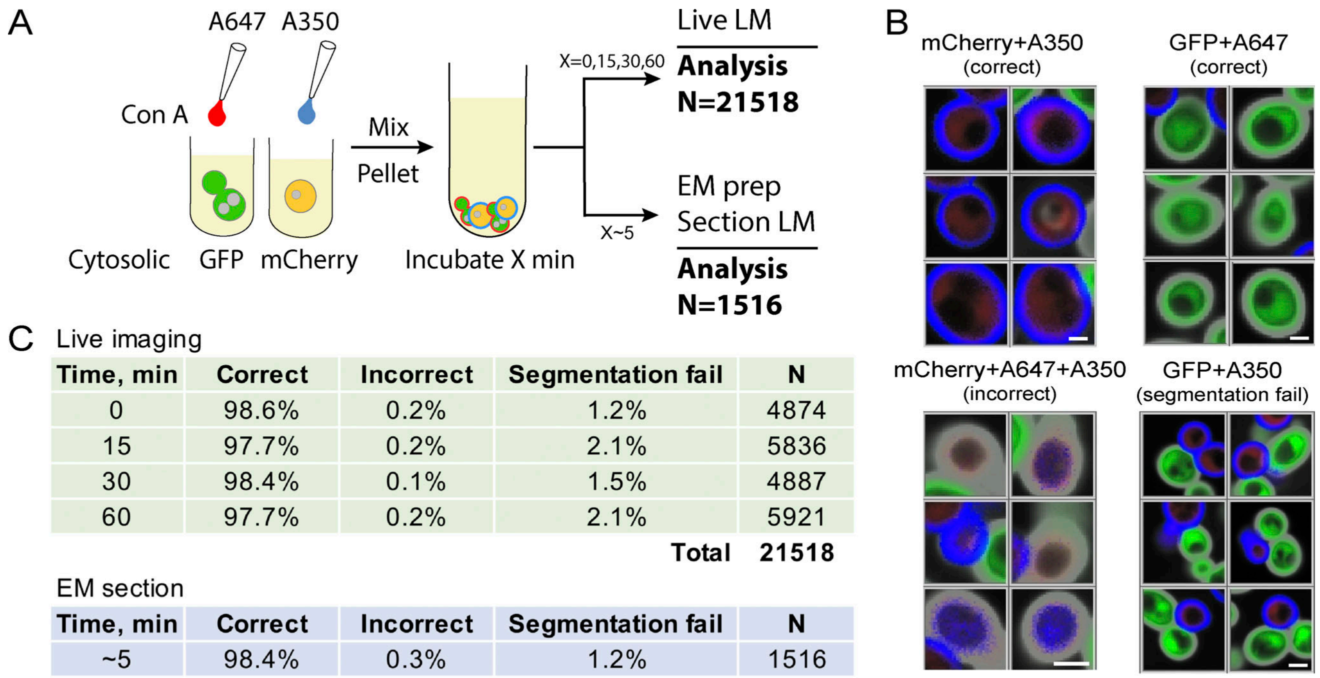

Live imaging

Time, min	Correct	Incorrect	Segmentation fail	N
0	98.6%	0.2%	1.2%	4874
15	97.7%	0.2%	2.1%	5836
30	98.4%	0.1%	1.5%	4887
60	97.7%	0.2%	2.1%	5921
			Total	21518

EM section

Time, min	Correct	Incorrect	Segmentation fail	N
~5	98.4%	0.3%	1.2%	1516

Figure S1. **MultiCLEM barcode verification. (A)** Assessment of the propensity to lose the correct barcode by Con A exchange between cells in the pellet during sample preparation. Cells expressing cytosolic GFP were labeled with Con A Alexa 647; cells with cytosolic mCherry were labeled with Con A Alexa 350. The cells were mixed, incubated as a pellet for 0–60 min, and analyzed by LM as living cells or after EM sample prep. **(B)** Examples of cells from the Con A exchange experiments (live-cell imaging) compiled from Scan^R Analysis software displayed as an overlay of Alexa 350 signal (blue), GFP signal (green), mCherry signal (red), and Alexa 647 signal (white). Scale bars: 1 µm. Top two panels present examples of correctly barcoded cells that are positive for mCherry and Alexa 350 or for GFP and Alexa 647. Bottom left panel shows examples of cells positive for mCherry and both Alexa 350 and 647, representing cells that acquired a second Con A stain and thus have an incorrect barcode. Bottom right panel shows examples of cells in which the image centers do not coincide with cell centers, indicating faulty segmentation during image analysis. This leads to an incorrect barcode assignment that would be excluded at the quality control step. **(C)** Proportions of cells with correct barcodes, incorrect barcodes, and wrongly segmented cells for live-cell imaging after different pellet incubation times and for imaging of EM sections. N, total number of analyzed cells in each row.

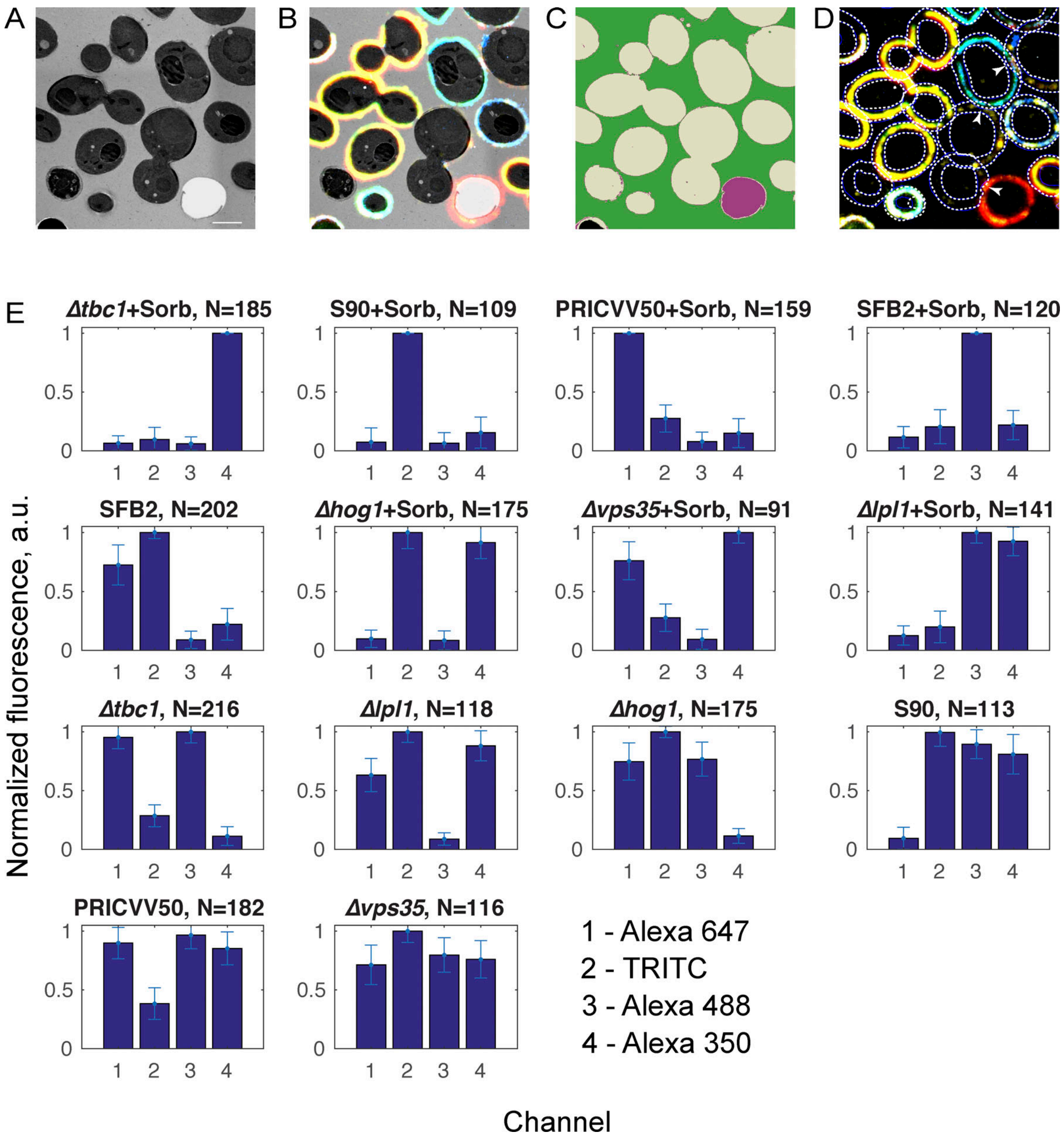

Figure S2. **Automated image processing and barcode assignment**. **(A–D)** Close-up view of part of the EM image shown in Fig. 3 A highlighting segmentation and fluorescence intensity measurements. **(A)** EM montage region. Scale bar: 2 µm. **(B)** The same region as in A, overlaid with the fluorescent signal. **(C)** Ilastik segmentation of the same EM image. Cells suitable for imaging are shown in cream, resin is in green, holes in the resin are in purple, and electron-dense regions are in black. **(D)** Cell wall outlines determined from EM image segmentation and used to measure fluorescent intensities are shown in white dotted lines and overlaid with the composite fluorescent image; white arrowheads show some of the regions excluded from masks due to intersections with neighboring cell walls. **(E)** Mean normalized fluorescence intensities for each barcode identified by k-means classification of all the cells in the osmotic shock experiment, showing all 14 combinations of fluorophores used in the experiment assigned to corresponding experimental condition. Error bars show SDs confirming the uniformity of each barcode.

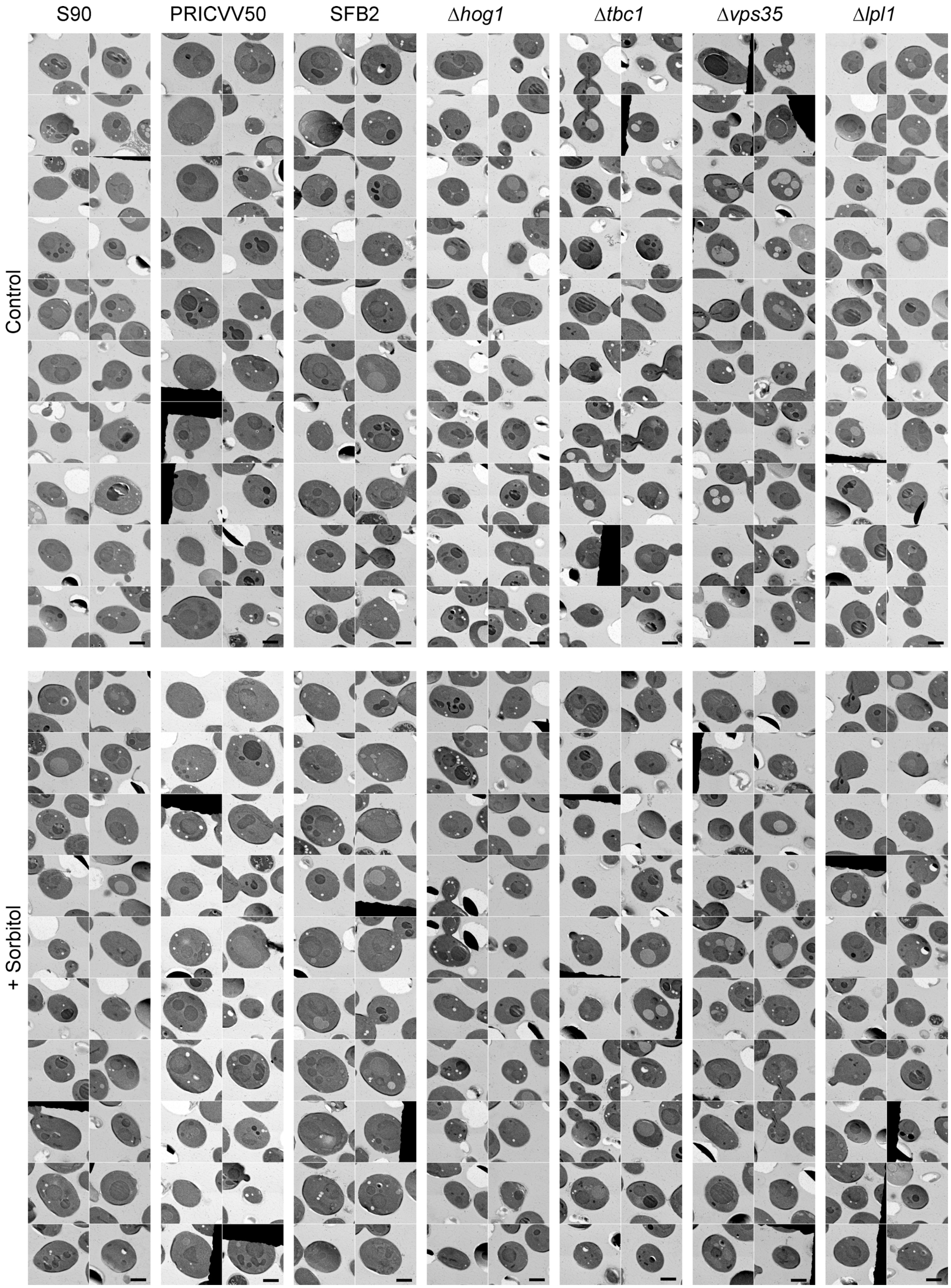

Figure S3. **Gallery of yeast cell sections from different strains and experimental conditions.** 20 random examples of images from the final high-resolution EM dataset are shown for each strain. No quality control or removal of poorly preserved cells was performed on this dataset to allow the reader to assess preservation quality. All cells display good preservation. A larger maximal cell cross section area is apparent for wine yeast. Scale bars: 2 µm.

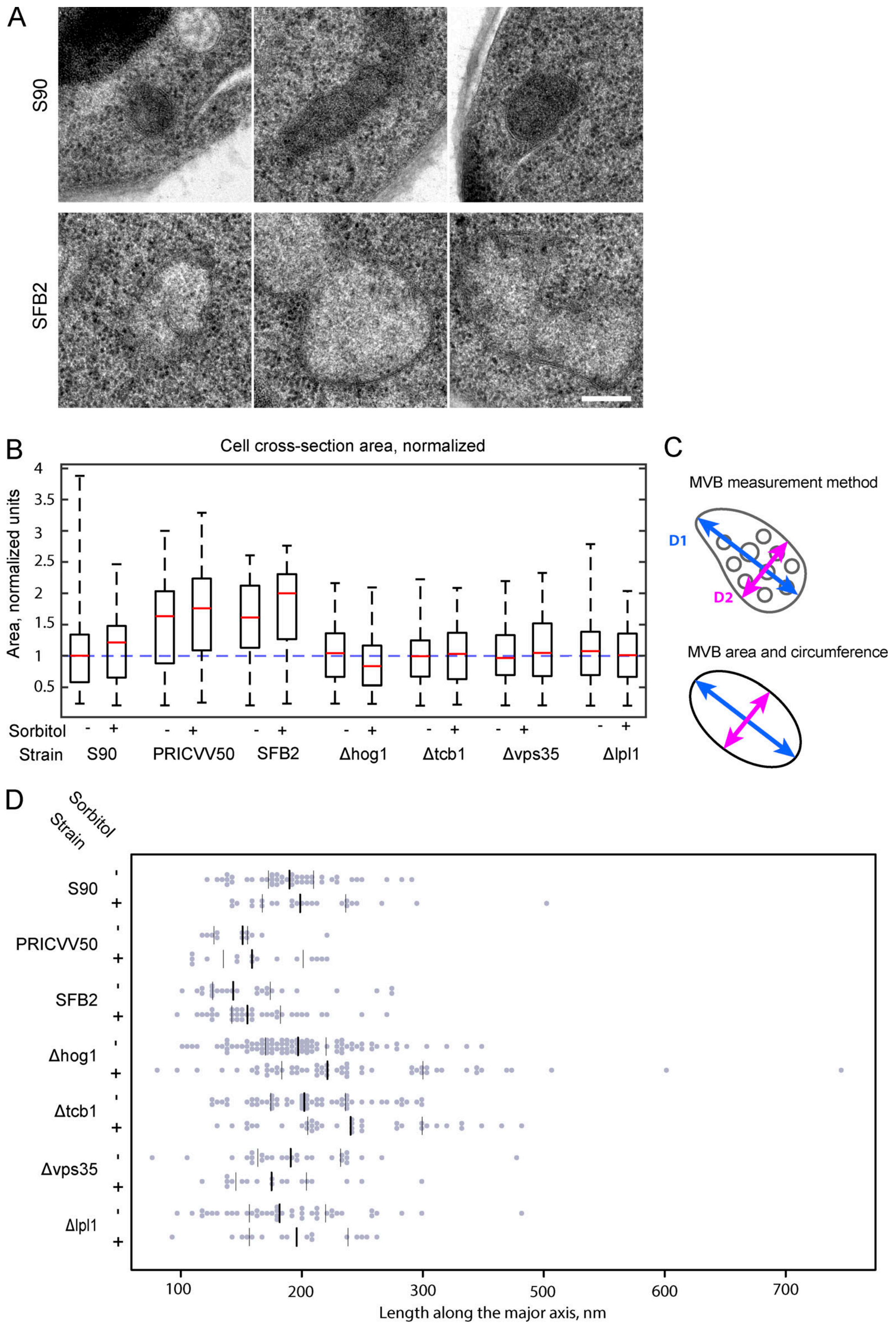

Figure S4. **Ultrastructural variability of the yeast strains under normal and osmotic shock conditions. (A)** Examples of mitochondria with normal morphology from the S90 strain (top) and abnormal swollen mitochondria from SFB2 wine yeast (bottom) under normal osmotic conditions. **(B)** Distributions of total mean cell cross section areas for different strains and conditions, normalized to the S90 control (blue dashed line); boxes show the 25th, 50th, and 75th percentiles, whiskers show the range. PRICVV50 and SFB2 strains were characterized by increased cross section areas, which signifies larger cells. PRICVV50 is a diploid (Novo et al., 2009), which explains their larger cell size, suggesting that SFB2, which has a similar size distribution, is also a diploid. A total of 1,748 cross sections was analyzed. **(C)** The area of MVB cross sections was measured not by point counting as in regular stereology but by approximation with an ellipse of similar shape: MVB major and minor axes (D1, D2) were determined (top), and area and circumference of an ellipse with the same axes were calculated (bottom) and used as an approximation of cross section area and circumference of the measured MVB. See Materials and methods for details. **(D)** Individual measurements of all MVB cross sections' major axes in all strains and conditions. Bold lines indicate the mean; thin lines indicate SD. Scale bar: 200 nm.

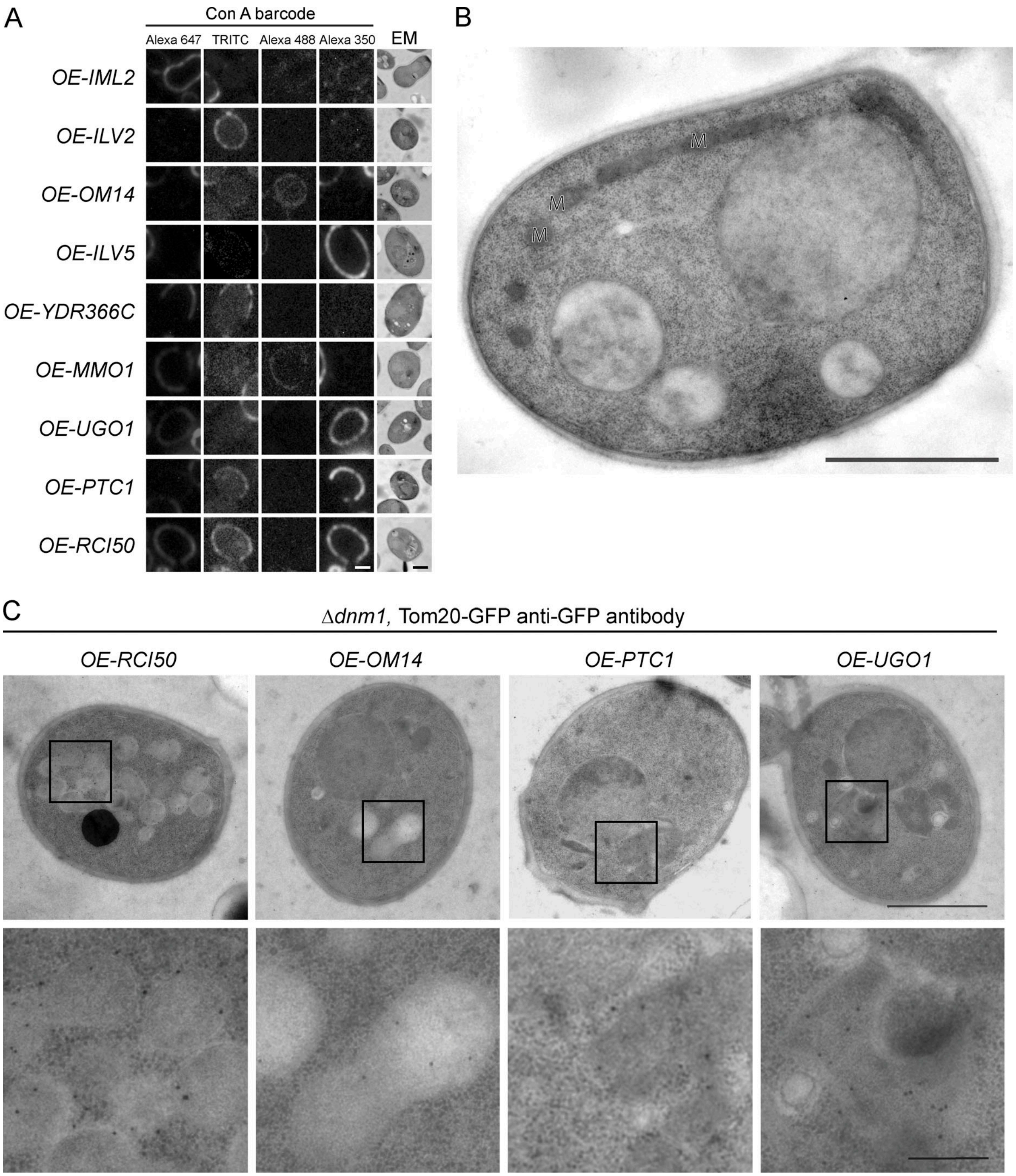

Figure S5. **Mitochondrial morphology studied by MultiCLEM. (A)** Examples of Con A barcodes for the mitochondria morphology experiment. First four columns show the LM images of the cell in different channels, fifth column shows EM image of the same cell. The name of the overexpressed (OE) gene is shown on the left for each strain. All strains are on the Δ*dnm1* background. **(B)** EM image of a WT cell without immunolabeling. M, mitochondria. **(C)** Four examples of Δ*dnm1* strains expressing *TOM20-GFP* and overexpressing proteins Rci50, Om14, Ptc1, and Ugo1. All samples were labeled with anti-GFP antibodies. Top row, full cell view; bottom row, close-up view of the regions marked by squares, showing immunogold labeling. Based on texture, bounding membrane morphology, and labeling pattern, compartments labeled in the *OE-OM14* and *OE-UGO1* strains are likely to be vacuoles, while the structures in *OE-PTC1* and *OE-RCI50* may be mitochondria with an unusual morphology. Scale bars: 1 µm in A and B (top), 200 nm in B (bottom).

https://doi.org/10.1083/jcb.201812081

Table S2. **Fluorescent microscopes and filters used in this study**

Characteristic	Zeiss Cell Observer HCS	Zeiss Cell Observer Z1	Nikon TE2000
Objective	63×, oil immersion	I Plan-Apochromat 63×/1.4, oil immersion	Plan Apo 60× oil
Alexa 350 filter set	335–383, 395, 420–470	365, 395, 395–495	–, 400dclp, –
Alexa 488 filter set	450–490, 495, 500–550	430–510, 495, 475–575	450–490, 495, 500–550
TMR filter set	553–577, 585, 594–646	525–575, 570, 535–675	530–560, 570LP, 590–650
Alexa 647 filter set	625–655, 660, 665–715	610–670, 660, 640–740	590–650, 660, 663–737
Cy7 filter set			721–749, 757LP, 770–850
Light source	X-cite 120LED	HXP120	Niji LED

For each filter set, excitation filter transmission range, dichroic mirror splitting wavelength, and emission filter transmission range are given in nanometers.

Table S1 is provided online as an Excel file and provides a list of all yeast strains used in this study. A supplemental PDF describes the yeast fluorescent barcoding protocol.

Reference

Novo, M., F. Bigey, E. Beyne, V. Galeote, F. Gavory, S. Mallet, B. Cambon, J.-L. Legras, P. Wincker, S. Casaregola, and S. Dequin. 2009. Eukaryote-to-eukaryote gene transfer events revealed by the genome sequence of the wine yeast Saccharomyces cerevisiae EC1118. *Proc. Natl. Acad. Sci. USA.* 106:16333–16338. https://doi.org/10.1073/pnas.0904673106
