# Supplementary material for: High-throughput ultrastructure screening using electron microscopy and fluorescent barcoding
Source: J Cell Biol. 2019 Jul 9;218(8):2797–811. doi: 10.1083/jcb.201812081 (PMC6683748; doi:10.1083/jcb.201812081)
Supplement: Data S1 (PDF) [file JCB_201812081_DataS1.pdf]

Supplementary Data 1. Yury. S. Bykov, Nir Cohen et al. 2018

The yeast fluorescent barcoding protocol

Materials and equipment

- Yeast media
- Phosphate buffered saline (PBS)
- Concanavalin A (Con A) storage stock solutions, 2.5 mg/ml in PBS. Prepare from commercially available lyophilized powder, sonicate to dissolve, aliquot and store at -20˚C only. 50-100 µl aliquots are usually a good choice.
- Tubes or plates with gas-permeable membrane
- Spectrophotometric cuvettes (plastic)
- Fresh YPAD agar plate
- Razor blade
- 0.3 mm and 0.1/0.2 mm aluminum membrane carriers for HPM010
- Tweezers and forceps
- Small flat metal spatula (0.5 cm wide)
- Pipette (0.5-10 µl)
- MILLIPORE filtering setup with 0.45 um filter
- Spectrophotometer
- Yeast incubator
- Ultrasonic bath
- Centrifuge for plates (e.g. Eppendorf 5820R)
- Standard benchtop centrifuge for 1.5 ml tubes
- (Optional) Ultracentrifuge and rotor, 1.5 ml ultracentrifuge tubes (e.g. Beckman Optima Max with TLA55 rotor)
- HPM010 high-pressure freezing machine

Yeast cultures

For freezing 3-5 HPM010 carriers the total amount of yeast cells should be 15-20 ml at OD_{600}=0.6. This means that for a 15-fold multiplexed experiment each strain should be grown in 1 ml to OD_{600}=0.6. This can be done in separate tubes or in a 24-well plate. Separate tubes are more difficult to handle but easier to grow (in a normal incubator). 24-well plates require smaller amplitude and higher speed orbital shaker (they cannot be properly mixed with a normal incubator for flasks). One can do it by using a plate shaker, or by sticking a plate to the top of the Eppendorf Thermomixer which is in turn placed inside a regular incubator at 30°C. The cultures can be prepared the day before to reach the OD=0.6 in the morning, so staining can be started immediately, or grown from diluted overnight cultures during the day. We use the following growth protocol to achieve desired cell density before freezing in the afternoon.

1. Dilute the overnight cultures in tubes or in a plate. To get OD=0.6 at 14:00 one would need an OD of approximately 0.15 at 09:30 for relatively fast growing strains.

2. If using a plate, seal it with a gas-permeable membrane, and install on the thermomixer or plate shaker inside the incubator at 30°C. The shaking speed should be 500-600 rpm.

Staining solution preparation

Staining solutions are prepared in two steps to reduce the errors and speed up the process. First concentrated 5X Con A stocks are prepared from the frozen Con A storage stocks. Relative brightness of different fluorescent channels can be adjusted at this stage. Then 5X stocks are mixed in equal amounts to produce the final staining solutions.

1. Thaw Con A storage stock solutions (2.5 mg/ml in PBS) and sonicate for 5 minutes in an ultrasonic bath to dissolve the aggregates.
2. Spin down any remaining large aggregates on a tabletop centrifuge at maximum speed, (16000 rcf), 4°C.
3. Prepare 5X stock solutions for fast mixing. To compensate for different conjugate brightness the concentrations can be adjusted at this stage (Table 1).
4. Optional: ultracentrifuge 5X stocks 30 min at 200000 g (45000 rpm) on TLA55 rotor of Beckmann tabletop centrifuge to remove more aggregates.
5. Mix the 5X stocks with each other and with PBS according to the pattern shown in Table 2 to get final staining solutions[1]. To increase speed and accuracy of mixing, label the tube rims in advance using markers of different colors showing which 5X stocks are added to which tube. First add the blue 5X stock to all tubes with the blue mark, then the green 5X stock to all tubes with the green mark etc.

Table 1. Example of 5X Con A stocks preparation recipe. The amount of Con A storage stock can be easily increased if it starts going bad, or more labelling intensity is needed without adjusting the final stock preparation.

Conjugate name	Con A storage stock, µl	PBS, µl	Total, µl
**B: Con A Alexa 350**	120	930	1050
**G: Con A Alexa 488**	40	1010	1050
**O: Con A TMR 555**	50	1000	1050
**R: Con A Alexa 647**	100	950	1050
**IR: Con A Cy7 (home made)**	250	800	1050

[1] The scheme can easily be extended to 5 colors with the same total volume – 600 µl – by adding extra 120 µl of the fifth stock solution and reducing the amount of PBS accordingly.

Table 2. Final staining solutions preparation. For each staining solution the amount of each 5X stock (denoted by the first letter of color, see Table 1) and PBS to add is indicated in µl.

Solution	B	G	O	R	PBS
**1**	120	0	0	0	480
**2**	0	120	0	0	480
**3**	0	0	120	0	480
**4**	0	0	0	120	480
**5**	120	120	0	0	360
**6**	120	0	120	0	360
**7**	120	0	0	120	360
**8**	0	120	120	0	360
**9**	0	120	0	120	360
**10**	0	0	120	120	360
**11**	120	120	120	0	240
**12**	120	120	0	120	240
**13**	120	0	120	120	240
**14**	0	120	120	120	240
**15**	120	120	120	120	120

Staining and freezing

1. Check the OD_{600} of all or a representative subset of strains. It should be 0.5-0.6. When the right OD is achieved, start filling HPM010 with nitrogen before proceeding to the next steps. Filling takes 15-20 minutes.
2. Spin down the cells at 1500 rcf for 5 min.
3. Carefully remove the plate from the centrifuge and discard the supernatants without disturbing the pellets. The pellets are very loose at this stage! Immediately after removing the supernatant fill the well with the staining solution and mix well.
4. Make sure the pellets are resuspended well and put the plate on the mixer in the incubator for 5-10 minutes. Avoid exposing to bright light.
5. Remove the plate from the incubator and spin it down at 1500 rcf for 5 min. Discard the supernatants and resuspend pelleted cells in medium. The pellets will cover the whole well bottom and may require thorough mixing in order to collect and resuspend all of the cells.
6. During the resuspension or afterwards pool all resuspended pellets in one 50 ml falcon tube. Vortex it well.
7. Proceed immediately. Collect the yeast on a filter using the MILLIPORE filtering device, and immediately put the filter on a fresh YPAD plate. Make sure it is wetted by the plate and that there are no bubbles in-between the filter and the agar surface.
8. Scrape the cell slurry on the filter into a dense clump with a spatula. The cells should be as concentrated as possible, but not too much: the concentration is too high and cells need diluting if after placed into HPM010 carrier, the slurry droplet surface quickly becomes opaque. To make the slurry more dilute, press filter with a spatula to squeeze water from the plate. To concentrate the cells lift the filter for a short time to make the cells dry a little bit.
9. Cut off the end of the pipet tip to make the hole wider, pipette 1.5-1.75 µl of suspension into the tip, holding the cell clump with a spatula. Do not press too hard to avoid diluting the suspension.
10. Apply the suspension to the 0.1 mm cavity of a high-pressure freezer membrane carrier, cover with a flat side of a 0.3 mm cavity carrier, and freeze immediately.
